# The Role of Maternally Acquired Antibody in Providing Protective Immunity Against Nontyphoidal *Salmonella* in Urban Vietnamese Infants: A Birth Cohort Study

**DOI:** 10.1093/infdis/jiy501

**Published:** 2018-10-12

**Authors:** Ruklanthi de Alwis, Le Thi Phuong Tu, Nhi Le Thi Quynh, Corinne N Thompson, Katherine L Anders, Nguyen Thi Van Thuy, Nguyen Trong Hieu, Lu Lan Vi, Nguyen Van Vinh Chau, Vu Thuy Duong, Tran Thi Hong Chau, Ha Thanh Tuyen, Tran Vu Thieu Nga, Pham Van Minh, Trinh Van Tan, Trang Nguyen Hoang Thu, Tran Do Hoang Nhu, Guy E Thwaites, Cameron Simmons, Stephen Baker

**Affiliations:** 1 Oxford University Clinical Research Unit, Hospital for Tropical Diseases, Wellcome Trust Major Overseas Programme, Ho Chi Minh City, Vietnam; 2 Hung Vuong Hospital, Ho Chi Minh City, Vietnam; 3 Hospital for Tropical Diseases, Ho Chi Minh City, Vietnam; 4 Centre for Tropical Medicine, Nuffield Department of Clinical Medicine, Oxford University, Oxford, United Kingdom; 5 London School of Hygiene and Tropical Medicine, London, United Kingdom; 6 Department of Medicine, University of Cambridge, Cambridge, United Kingdom; 7 Program in Emerging Infectious Diseases, Duke University–National University of Singapore (Duke-NUS) Medical School, Singapore; 8 Viral Research and Experimental Medicine Centre, SingHealth Duke-NUS Academic Medical Centre, Singapore; 9 School of Biological Sciences, Monash University, Victoria, Australia; 10 Department of Microbiology and Immunology, University of Melbourne, Melbourne, Australia

**Keywords:** Nontyphoidal *Salmonella*, NTS, Vietnam, *Salmonella* Typhimurium, *Salmonella* Enteritidis, maternal antibodies, infant antibodies, seroincidence, seroepidemiology, transplacentally acquired immunity, maternal immunization

## Abstract

**Background:**

Nontyphoidal *Salmonella* (NTS) organisms are a major cause of gastroenteritis and bacteremia, but little is known about maternally acquired immunity and natural exposure in infant populations residing in areas where NTS disease is highly endemic.

**Methods:**

We recruited 503 pregnant mothers and their infants (following delivery) from urban areas in Vietnam and followed infants until they were 1 year old. Exposure to the dominant NTS serovars, *Salmonella enterica* serovars Typhimurium and Enteritidis, were assessed using lipopolysaccharide (LPS) O antigen–specific antibodies. Antibody dynamics, the role of maternally acquired antibodies, and NTS seroincidence rates were modeled using multivariate linear risk factor models and generalized additive mixed-effect models.

**Results:**

Transplacental transfer of NTS LPS–specific maternal antibodies to infants was highly efficient. Waning of transplacentally acquired NTS LPS–specific antibodies at 4 months of age left infants susceptible to *Salmonella* organisms, after which they began to seroconvert. High seroincidences of *S*. Typhimurium and *S*. Enteritidis LPS were observed, and infants born with higher anti-LPS titers had greater plasma bactericidal activity and longer protection from seroconversion.

**Conclusions:**

Although Vietnamese infants have extensive exposure to NTS, maternally acquired antibodies appear to play a protective role against NTS infections during early infancy. These findings suggest that prenatal immunization may be an appropriate strategy to protect vulnerable infants from NTS disease.

## Teaser

We found a high transfer ratio of anti-nontyphoidal *Salmonella* (NTS) antibodies from mothers to infants. Furthermore, despite a high seroincidence of NTS in infants, maternally acquired antibodies provided protection from seroconversion. Therefore, we propose prenatal immunization against NTS as a possible strategy for protecting infants from NTS disease.

Nontyphoidal *Salmonella* (NTS) organisms are a significant cause of diarrhea and bloodstream infections globally [1]. In 2010, gastroenteritis-associated NTS bacteria caused an estimated 93.8 million cases and 155000 deaths globally, with the highest burden in children of developing countries [2]. More-severe, extraintestinal NTS infections, known as invasive NTS disease, occurred in approximately 3.4 million individuals globally. The estimated case-fatality rate of invasive NTS disease was 20% in 2010, with 63.7% of fatal cases occurring in children aged <5 years of age [3]. Rising levels of antimicrobial resistance are likely to compound invasive NTS disease morbidity and mortality. *Salmonella enterica* serovars Typhimurium and Enteritidis are the first- and second-most commonly isolated *Salmonella* from humans worldwide, respectively [4], constituting >60% of all *Salmonella* bacteria isolated from humans [4]. Consequently, bivalent *S*. Typhimurium and *S*. Enteritidis vaccine formulations are a current focus for development of a human NTS vaccine [5]. However, a lack of accurate data regarding natural exposure and infant immunity to NTS has impaired evidence-based recommendations for vaccine development, scheduling, and implementation.

Antibodies play a critical role in protective immunity against NTS infections. Several outer-membrane *Salmonella* antigens, including lipopolysaccharide (LPS; which encompasses the O antigen), are targets of human antibodies following natural infections [6, 7]. NTS-specific immunoglobulin A (IgA) antibodies in the gut have been shown to reduce bacterial entry into epithelial cells [8], whereas NTS-specific immunoglobulin G (IgG) antibodies in the blood have been shown to protect against invasive NTS disease through cell-mediated mechanisms and complement membrane attack complexes [9, 10]. For many infectious pathogens, maternally acquired antibodies transferred during pregnancy or through breast milk provide an important additional layer of protection during the infant’s first months of life [11–15]. In the case of NTS infections, several studies using animal models have indicated a potential role for placentally transferred maternal NTS-specific IgG and breast milk IgA in protecting newborns [16, 17]. Similarly, studies from Malawi have suggested that a lack of symptomatic NTS infections in the first months of infant life may be associated with the transfer and persistence of maternal antibodies [9, 18]. However, given the paucity of available longitudinal maternal and infant data, it is unclear whether maternally acquired immunity plays a significant protective role against NTS organisms in a low-to-middle-income setting where NTS is highly endemic.

The transfer, longevity, and role of anti-NTS maternal antibodies in infants have not been defined. Furthermore, because of underreporting and subclinical disease, the true burden of NTS infection and exposure in infants is difficult to accurately define. NTS infections in children represent a large public health burden in Vietnam, where 4% of pediatric hospitalized diarrheal disease cases and 18% of infant diarrheal disease cases in the community have been reported to be associated with NTS [19, 20]. We aimed to assess the dynamics of anti-*Salmonella* antibody in the first year of life in an urban cohort of infants in Ho Chi Minh City (HCMC), Vietnam. We measured the transfer and decay of anti-NTS maternal antibodies in infants, estimated the true incidence of NTS exposure (through the progression of the *S.* Typhimurium and *S*. Enteritidis O antigen IgG response), and finally assessed the potential of transplacentally acquired antibodies in protecting infants against NTS infections.

## METHODS

### Study Design and Ethics

The current study was a component of a larger prospective birth cohort study conducted in Vietnam, for which a detailed description has been published previously [19, 21]. Mothers and infants were participants from the mentioned birth cohort study who were recruited between January and December 2013 at Hung Vuong Obstetrics Hospital in HCMC. Briefly, healthy pregnant woman aged >15 years who were HIV seronegative and living in an urban district (District 8) of HCMC were invited to participate in the study. Umbilical cord and venous maternal blood samples were collected immediately following delivery. Infants were enrolled into the study after birth, and baseline information related to the mother and the infant’s health after delivery was recorded within 72 hours after birth. After delivery, infants were called back regularly for routine follow-up visits until 1 year of age, with blood samples collected at 4- or 6-month visits, 9-month, and 12-month visits. All blood samples were separated into cells and plasma and stored at −20^°^C until further use. This study was approved by the Institutional Review Board of Hung Vuong Obstetric Hospital and the Oxford Tropical Research Ethics Committee (OxTREC). All participating mothers provided written informed consent for themselves and their infants.

Plasma and stool samples for validation of O antigen–specific enzyme-linked immunosorbent assay (ELISA) findings were obtained from a previously published study [22]. Multiplex polymerase chain reaction (PCR) analyses were conducted on stool samples culture positive for *Salmonella*, for identification of the infecting *Salmonella* serovars. Further details are available in the Supplementary Materials and Supplementary Table 1.

### *S*. Typhimurium and *S*. Enteritidis O Antigen ELISAs

ELISAs were used to quantify plasma antibodies targeting O antigen of *S.* Typhimurium (O4) and *S.* Enteritidis (O9), as previously described for *S.* Typhi [23, 24]. Briefly, ELISA plates were coated with Toll-like receptor–grade smooth form of LPS (Enzo Life Sciences, UK) from *S.* Typhimurium or *S.* Enteritidis at 0.5 mg/mL and incubated overnight at 4°C. Plates were blocked with 5% nonfat dried milk in phosphate-buffered saline for 2 hours at room temperature, and incubated with plasma samples (1:200 diluted in 1% nonfat dried milk buffer) for 2 hours at room temperature. LPS-binding antibodies were detected using alkaline phosphatase-conjugated anti-human IgG and immunoglobulin M (IgM) secondary antibodies (for 1 hour at ambient room temperature), and developed using p-nitrophenyl-phosphate solution (SigmaFAST N1891; Sigma-Aldrich, UK), with absorbance measured at 405 and 490nm. The OD of each sample was then converted to ELISA units (EU) per milliliter, using a reference standard as previously described [24]. The reference standard on each plate contained ten 2-fold dilutions, starting from a 1:200 dilution. A standard curve was generated from corresponding ODs, using a 4-parameter logistic regression fit, where 1 EU was the reciprocal of the dilution of the reference standard that gave an OD of 1. The reference standard was a pool of known anti-O antigen–positive human sera generated from a bank of Vietnamese plasma.

### Serum Bactericidal Assay

A selection of infant plasma samples with high (>1000 EU; n = 11) and low (<50 EU; n = 9) binding antibody titers were tested for bactericidal killing activity against a Vietnamese clinical isolate of *S.* Typhimurium (VNS20081; isolated from a Vietnamese child with diarrhea [22]), using a previously described procedure [25]. Briefly, heat-inactivated plasma samples were serially diluted 2-fold from 1:50 to 1:6400 in phosphate-buffered saline containing 1% bovine serum albumin, 33 mg/mL CaCl_2_, and 0.041% MgCl_2_.6H_2_O. *S.* Typhimurium was grown to mid-log phase (OD = 0.4) and diluted to approximately 250–300 colony-forming units (CFU)/well. Baby rabbit complement was added to a final concentration of 25% and incubated at 37°C for another 60 minutes. *S.* Typhimurium bacteria were enumerated at time 0 (T0) and after incubation for 60 minutes (T60) by plating on nutrient agar plates. Bactericidal activity was expressed as a ratio between T60 and T0 (calculated as the number of CFU at T60 divided by the number at T0), and the serum bactericidal titer at 50% bactericidal effect was calculated. Each plasma sample was tested in triplicate and averaged.

### Statistical Analysis

Comparisons between groups of continuous data were conducted using the nonparametric test, Mann-Whitney *U* test. Comparisons between continuous data of paired samples were conducted using the paired Wilcoxon test. After the normality of the IgG titer distribution was tested using the Shapiro-Wilk normality test, correlation between newborn and maternal IgG titers were tested using the Pearson correlation test. Seroincidence rates were calculated as the number of seroconversions divided by the number of infant-years, multiplied by 1000, where seroconversion was defined as at least a 2-fold increase in IgG titer. Seroincidence rates between periods were compared using the Wald test.

Risk factors affecting the transfer ratio of anti-O4 and anti-O9 IgG (calculated as the neonatal IgG titer divided by the maternal IgG titer) were assessed using a linear regression model, with the log_10_-transformed IgG transfer ratio as the dependent variable. Thirteen variables (Tables 2 and 3) were chosen on the basis of past evidence or the potential for affecting maternal-infant IgG transfer [26, 27]. Univariable linear regression was followed by multivariable regression modeling with these 13 variables and backward stepwise elimination using the Akaike information criterion (AIC) as best-fit criteria.

Time series data of anti-O4 and anti-O9 IgM and IgG titers in infants during the 12-month study follow up period were fitted to generalized additive mixed-effect models (GAMM), with adjustments for random intercepts and slopes. Smooth, nonlinear GAMMs were fitted to the data by using the R packages *mgcv* and *itsadug*. Because the log_10_ transformation of 0 yields a value of infinity, for modeling and graphical purposes data points with values of 0 EU were substituted with a small value (ie, 0.00001).

## RESULTS

### O Antigen Antibodies Identify *S*. Typhimurium and *S*. Enteritidis Infections

A rise in titers of IgM and IgG antibodies specific to *S*. Typhimurium O4 and *S*. Enteritidis O9 antigens is indicative of a recent infection with *Salmonella* bacteria [28, 29]. To confirm that such a rise was also evident in Vietnamese children, we conducted a series of ELISAs on paired plasma samples (obtained during the acute and convalescent phases) collected during a prior study [22] from children residing in HCMC and presenting with acute diarrhea associated with culture-confirmed *S*. Typhimurium (n = 36), *S*. Enteritidis (n = 6), other group B (O4) *Salmonella* (n = 20), other group D (O9) *Salmonella* (n = 2), and *Shigella* (n = 31) infections. In these patient samples, both anti-O4 IgM and IgG exhibited a significantly greater median fold increase after infection with *S*. Typhimurium (4.2-fold and 4.9-fold, respectively) than infection with non–group B NTS (1.0-fold and 0.9-fold, respectively) or *Shigella* organisms (1.2-fold and 1.04-fold, respectively; Supplementary Figure 1*A* and 1*B*). Similarly, we observed an increase in anti-O9 IgM and IgG titers between paired samples following infection with *S*. Enteritidis (2.4-fold and 3.1-fold, respectively) and other group D *Salmonella* organisms (6.3-fold and 9.0-fold, respectively; Supplementary Figure 1*C* and 1*D*). Our data show that these O antigen ELISAs were sufficiently specific to detect and differentiate infection with group B and group D *Salmonella* bacteria from infection with non-NTS organisms.

### Maternal Transfer of *Salmonella*-Specific Antibodies to Newborns

We enrolled 503 pregnant mothers with a median age of 28 years (interquartile range, 25–31 years) and enrolled their infants following delivery (Table 1). Approximately 9% of enrolled mothers (45 of 503) had complications during pregnancy, 7% (34 of 503) experienced hypertension, and 4.4% (22 of 503) were diabetic. Among the enrolled infants, the sex of 52% (260 of 503) was male, 4.2% (21 of 503) were preterm, and 4.6% (23 of 503) had a low birth weight. We performed anti-O4 and anti-O9 IgG ELISAs on plasma and placental cord blood specimens from 503 participating mothers following delivery. The anti-O4 IgG geometric mean titers were 191 EU/mL (95% confidence interval [CI], 178–204) in participating mothers and 252 EU/mL (95% CI, 234–271) in infants at birth (Table 1). Similarly, the maternal and infant anti-O9 IgG geometric mean titers were 160 EU/mL (95% CI, 149–172) and 177 EU/mL (95% CI, 163–193), respectively.

**Table 1.** Baseline Characteristics of 503 Participating Mother-Infant Pairs

Characteristic	Value
Mothers	
Age, y	28 (25–31)
Education level, higher secondary or above	248 (49.3)
Gravidity	2 (1–3)
Pregnancy complication	45 (8.95)
Fever from infection during pregnancy	24 (4.8)
Diabetes	22 (4.4)
Hypertension	34 (6.8)
Anemia	34 (6.8)
Smoking or passive smoking during pregnancy	194 (38.6)
IgG level, EU/mL, GMT (95% CI)	
To anti-O4 antigen	191 (178–204)
To anti-O9 antigen	160 (149–172)
Infants	
Male sex	260 (51.7)
Gestation age, wk	39 (38–40)
Preterm birth	21 (4.2)
Birth weight, kg	3·15 (2.90–3.40)
Low birth weight	23 (4.6)
Vaginal delivery	288 (57.3)
Exclusive breastfeeding	
During first month of life	215 (43)
During first 4 months of life	93 (18)
IgG level, EU/mL, GMT (95% CI)	
To anti-O4 antigen	252 (234–271)
To anti-O9 antigen	177 (163–193)

Data are no. (%) of participants or median value (interquartile range), unless otherwise indicated.

Abbreviations: CI, confidence interval; GMT, geometric mean titer; IgG, immunoglobulin G.

**Figure 1.**
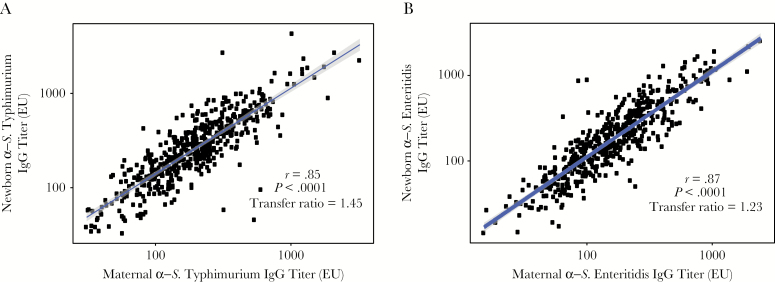
High transplacental transfer of anti-*Salmonella enterica* serovar Typhimurium antibodies (ie, O4-specific immunoglobulin G [IgG]) and anti-*S. enterica* serovar Enteritidis antibodies (ie, O9-specific IgG) from mothers to newborns. Titers of anti-*S*. Typhimurium–specific IgG (*A*) and anti-*S*. Enteritidis–specific IgG (*B*) show statistically significant positive correlation between paired maternal blood and infant cord-blood samples, with a high transfer ratio (ie, maternal to infant IgG ratio). The correlation between log_10_-transformed newborn and maternal IgG titers was tested using the Pearson correlation test.

We next assessed maternal antibody transfer of anti- *S*. Typhimurium (anti-O4) and anti-*S*. Enteritidis (anti-O9) IgG from mothers to newborns. There was significant linear correlation between maternal IgG titers and infant titers for both O4 and O9 (correlation coefficients, 0.85 (*P* < .0001) and 0.87 (*P* < .0001), respectively; Figure 1). Maternal anti-O4 and anti-O9 IgG were efficiently transferred to newborns, with mean transfer ratios (calculated is the newborn IgG titer divided by the maternal IgG titer) of >1 (ie, 1.45 [95% CI, 1.39–1.51] and 1.23 [95% CI, 1.17–1.30], respectively). Furthermore, a positive correlation (r = 0.69; *P* < .0001) was observed between anti-O4 and anti-O9 IgG titers in the maternal samples (Supplementary Figure 2).

Risk factor analysis was conducted to identify maternal and infant variables associated with the transfer of anti-O4 and anti-O9 IgG antibodies from mothers to infants. A multivariate linear regression analysis with log_10_-transformed anti-O4 IgG transfer ratios identified several variables associated with maternal antibody transfer. These factors included a positive correlation with maternal hypertension (*P* = .027) and a weak positive correlation with female infants (*P* = .085), whereas maternal anti-O4 IgG titer, preterm birth, and irregular vaginal bleeding negatively correlated with the transfer ratio (*P* = .0006, 0.012, and 0.061, respectively; Table 2). A similar multivariable linear regression analysis conducted with log_10_-transformed anti-O9 IgG transfer ratios identified female children and maternal hypertension as also being positively associated with the anti-O9 IgG transfer ratio (*P* = .028 and *P* = .048, respectively); preterm birth and irregular vaginal bleeding showed only weak evidence for a negative association (Table 3).

**Table 2.** Linear Risk Factor Models for the Log_10_-Transformed Anti-O4 Antigen–Specific Immunoglobulin G (IgG) Transfer Ratio as a Function of Several Maternal and Neonatal Covariates

Variable	Univariate Analysis		Multivariate Analysis	
	β (95% CI)	*P*	Adjusted β (95% CI)	*P* ^a^
Maternal age, y	−0.001 (−.005–.002)	.436	…	
Maternal education level, higher secondary or above	0.01 (−.02–.04)	.592	…	
Maternal O4 antigen–specific IgG level, log_10_ transformed	−0.09 (−.14 to −.04)	.0003	−0.09 (−.14 to −.04)	.0006
Preterm birth, gestation <37 wk	−0.11 (−.20 to −.03)	.009	−0.11 (−.19 to −.02)	.012
Low birth weight, <2500 g	−0.04 (−.12 to .04)	.377	…	
Female neonatal sex	0.04 (.003 to .07)	.034	0.03 (−.004 to .06)	.085
Smoking or passive smoking during pregnancy	0.02 (−.02 to .05)	.295	…	
Irregular vaginal bleeding	−0.08 (−.16 to −.001)	.049	−0.07 (−.15 to .003)	.061
Diabetes	0.03 (−.05 to .12)	.414	…	
Intrauterine growth restriction	−0.01 (−.11 to .010)	.879	…	
Maternal hypertension	0.07 (.001 to .13)	.048	0.07 (.01 to .14)	.027
Maternal anemia	0.01 (−.06 to .08)	.789	…	
Fever from infection during pregnancy	0.003 (−.076 to .081)	.947	…	

Abbreviation: CI, confidence interval.

^a^For adjusted coefficients from the final multivariate risk factor model.

**Table 3.** Linear Risk Factor Models for Log_10_-Transformed Anti-O9 Antigen–Specific Immunoglobulin G (IgG) Transfer Ratio as a Function of Several Maternal and Neonatal Covariates

Variable	Univariate Analysis		Multivariate Analysis	
	β (95% CI)	*P*	β (95% CI)	*P* ^a^
Maternal age, y	−0.001 (−.004–.002)	.492	…	
Maternal education level, higher secondary or above	0.01 (−.02–.05)	.510	…	
Maternal O9 antigen–specific IgG level, log_10_ transformed	−0.001 (−.06 to .04)	.703	…	
Preterm birth, gestation <37 wk	−0.08 (−.17 to .001)	.056	−0.11 (−.19 to −.02)	.063
Low birth weight, <2500 g	−0.02 (−.10 to .06)	.558	…	
Female neonatal sex	0.04 (.005 to .07)	.024	0.03 (−.002 to .07)	.048
Smoking or passive smoking during pregnancy	−0.003 (−.04 to .03)	.874	…	
Irregular vaginal bleeding	−0.07 (−.15 to .01)	.105	−0.07 (−.15 to .01)	.086
Diabetes	0.04 (−.04 to .12)	.335	…	
Intrauterine growth restriction	−0.01 (−.12 to .09)	.785	…	
Maternal hypertension	0.07 (.004 to .14)	.037	0.08 (.01 to .14)	.028
Maternal anemia	0.01 (−.06 to .08)	.741	…	
Fever from infection during pregnancy	0.05 (−.03 to .13)	.230	…	

Abbreviation: CI, confidence interval.

^a^For adjusted coefficients from the final multivariate risk factor model.

### Seroconversion During the First Year of Life

**Figure 2.**
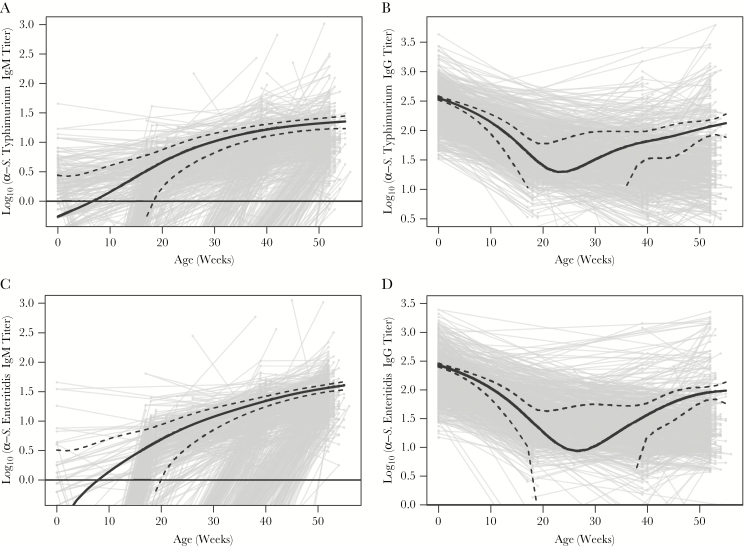
Antibody dynamics of *Salmonella enterica* serovar Typhimurium–specific antibodies (ie, O4) and *S*. *enterica* serovar Enteritidis–specific antibodies (ie, O9) during the first 12 months of life in infants from Ho Chi Minh City, Vietnam. Plots show a generalized additive mixed-effect model–fitted population trend (solid black line) for anti-O4 immunoglobulin M (IgM; *A*), anti-O4 immunoglobulin G (IgG; *B*), anti-O9 IgM (*C*), and anti-O9 IgG (*D*) from birth through 12 months of age. 95% confidence intervals are shown by the dashed black lines.

Enrolled newborns were followed-up for 12 months, and anti-*S*. Typhimurium (anti-O4) and anti-*S*. Enteritidis (anti-O9) IgM and IgG were measured at birth, 4 or 6 months, 9 months, and 12 months. The dynamics of antibody titers for anti-O4 and anti-O9 IgM and IgG over the first 12 months of life were investigated using GAMM models (Figure 2). The GAMM-fitted anti-O4 and anti-O9 IgM trend lines originated below the limit of detection and steadily increased at a slowing rate over the 12-month period (Figure 2A and C). Conversely, the anti-O4 and anti-O9 IgG trend lines declined over several months and then sporadically increased, likely reflecting exposure to O4- and O9-positive organisms (Figure 2B and 2D) . The lowest point of the GAMM-fitted IgG trend lines for anti-O4 and anti-O9 IgG was between 20 and 30 weeks of age.

Seroconversion was defined as a ≥2-fold increase in IgG titer against the respective O antigen. Over the follow-up period in our infant population, we observed 179 and 167 seroconversions to O4 and O9, respectively, with 120 infants seroconverting to both antigens, indicating some O antigen specificity (Supplementary Figure 3). Of the 503 enrolled infants, 359 were followed up for the 12-month period. We estimated the seroincidence of group B (most commonly *S*. Typhimurium) and group D (most commonly *S.* Enteritidis) *Salmonella* exposures during the first 12 months of infant life as 475 episodes/1000 infant-years (95% CI, 410–547) and 448 episodes/1000 infant-years (95% CI, 385–518), respectively (Table 4). We additionally observed that there were no O4 and O9 *Salmonella* seroconversions from 0 to 4 months of age, while the rate of seroconversion increased significantly between 5 and 9 months and again between 10 and 12 months (Table 4).

**Table 4.** Estimated Seroincidences of *Salmonella* Serogroup B and D Detection During the First Year of Life Among Infants in Ho Chi Minh City

*Salmonella* Serogroup	Seroincidence, Cases/1000 Infant-Years, Mean (95% CI)	*P*
Group B		
First year of life	475 (410–547)	
Age, mo		
0–4	0 (0–29)	
5–9	536 (418–677)	<.00001^a^
10–12	654 (487–860)	.276^b^
Group D		
First year of life	448 (385–518)	
Age, mo		
0–4	0 (0–29)	
5–9	474 (364–608)	<.00001^a^
10–12	847 (655–1077)	.0009^b^

Abbreviation: CI, confidence interval.

^a^By the Wald test, comparing incidence rates between ages 0–4 months and 5–9 months.

^b^By the Wald test, comparing incidence rates between ages 5–9 months and 10–12 months.

### Infants Protected Against Seroconversion to O Antigen by Maternally Acquired IgG

**Figure 3.**
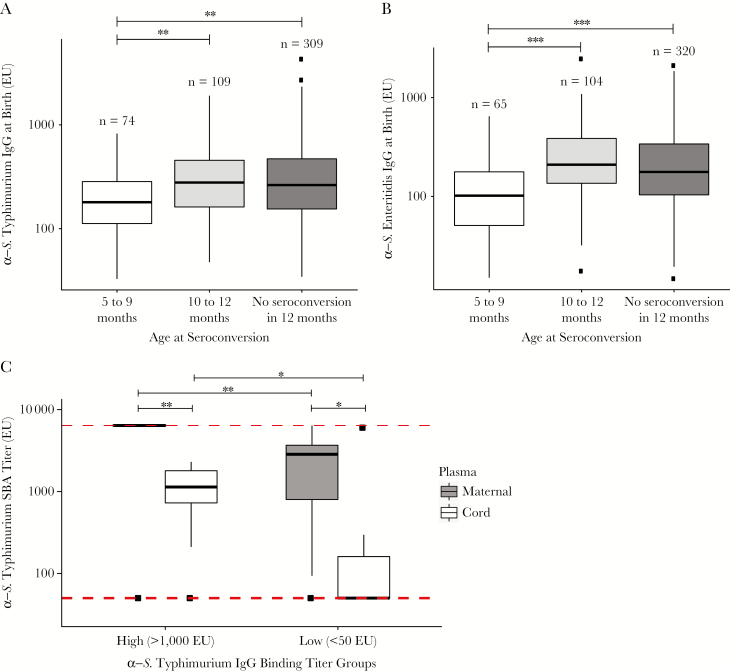
Protective role of maternally acquired nontyphoidal *Salmonella* (NTS)–specific immunoglobulin G (IgG) antibodies in infants. Seroconversion in younger age groups was associated with significantly lower *Salmonella enterica* serovar Typhimurium–specific IgG (ie, O4; *A*) and *S*. *enterica* serovar Enteritidis–specific IgG (ie, O9; *B*) titers at birth. *P > .01 but ≤ .05, **P ≥ .001 but ≤ .01, and ***P < .001, by the Mann-Whitney *U* test. *C*, Maternal plasma specimens with either high (ie, >1000 EU; n = 11) or low (ie, <50 EU; n = 9) anti-*S*. Typhimurium IgG binding titers (along with the paired cord plasma specimens) were tested for serum bactericidal assay (SBA) activity against a clinical isolate of *S*. Typhimurium. *P > .01 but ≤ .05, **P ≥ .001 but ≤ .01, and ***P < .001, by the paired Wilcoxon test, for comparison of SBA titers between paired maternal and cord plasma specimens, and by the Mann-Whitney *U* test, for comparison of SBA titers across high and low anti-*S*. Typhimurium IgG binding antibody titer groups. The red dashed lines at the top and bottom mark the maximum and minimum plasma dilutions, respectively, used in the assay. The numbers of maternal-infant pairs with high-binding and low-binding titers used in the assay were 11 and 9, respectively.

A lack of seroconversion during 0–4 months of age (Figure 2B and D) combined with an increasing seroincidence rate with age (Table 4) implied increased susceptibility of infants to *Salmonella* infections as a consequence of the decay of protective maternal antibodies or, alternatively, an increase in the exposure to these organisms after weaning. Infants who seroconverted at 10–12 months of age and those who did not seroconvert during the first 12 months of life had significantly greater anti-*S*. Typhimurium (anti-O4) and anti-*S*. Enteritidis (anti-O9) IgG titers at birth than those who seroconverted at 5–9 months of age (Figure 3A and B). Last, we measured the functional capacity of maternally acquired O4- and O9-specific antibodies to induce complement-mediated bacterial killing. In vitro serum bactericidal assays using a Vietnamese wild-type *S*. Typhimurium showed that paired maternal and infant cord plasma samples with high titers (>1000 EU) of anti-O4 IgG antibodies induced significantly greater serum bactericidal activity than those with low (<50 EU) anti-O4 IgG titers (Figure 3C). This observation indicates a protective role of maternally acquired antibodies during the early months of infant life and indicates a correlation between antibody titer and the ability to induce complement-mediated killing.

## DISCUSSION

We conducted a seroepidemiologic investigation of NTS-specific antibodies in a large number of mothers and their infants in the first year of life in urban Vietnam. Because mothers were recruited to the study prior to giving birth, we had the ability to collect data regarding complications during pregnancy and the mother’s condition prior to delivery. Additionally, the 1-year follow-up period allowed us to measure the longevity of maternal antibodies and to accurately determine the seroincidence of NTS in infants in HCMC. Our study defines the role and dynamics of NTS-specific maternal antibodies and better estimates the true burden of NTS infections in infants residing in a setting of endemicity.

Our study observed higher antibody concentrations of both anti-O4– and anti-O9–s––– pecific IgG in newborns than mothers at delivery. Similar observations have been observed with antibodies specific to other pathogens, such as influenza virus, measles virus, and *Bordetella pertussis* [14, 30, 31]. Because of the recent implementation of maternal vaccination to protect neonates from life-threatening infections, there has been considerable interest in factors affecting the transfer rates of antibodies to infants to optimize protection provided by prenatal vaccination [14, 32, 33]. Factors such as maternal antibody concentration [31, 34], IgG subtype [35, 36], antigen target [37], gestation age [35, 36] and pregnancy complications (such as hypertension) [38, 39] have been shown to affect transfer rates of antibodies specific to other antigens, such as measles virus, LPS (from *Klebsiella pneumoniae, Escherichia coli,* and *Pseudomonas aeruginosa*), tetanus toxoid, diphtheria toxoid, and pertussis antigens [26]. We identified maternal antibody levels and preterm birth to be negatively associated and maternal hypertension to be positively associated with the transfer ratio of anti-O4– and anti-O9–specific IgG. Negative associations between maternal antibody levels and transfer ratios have been observed previously and occur because of the saturation of the transplacental IgG transporter (ie, the fetal Fcγ receptor) in environments with high maternal IgG titers [26]. Despite this negative association, fetuses will still benefit from prenatal vaccination because children born to mothers with high IgG titers will also have high IgG concentrations.

A larger cohort, in whom the present study was nested, estimated the symptomatic diarrheal disease incidence in infants between 2009 and 2013 to be 89.4 cases/1000 infant-years, of which 18% (approximately 16.1 cases/1000 infant-years) involved stool specimens that were PCR positive for *Salmonella* [19, 21]. However, the true burdens of *S*. Typhimurium and *S*. Enteritidis infections (symptomatic and asymptomatic) in Vietnamese infants, as estimated here by the seroincidence, were significantly greater, at 475 cases/1000 Infant-years and 448 cases/1000 infant-years, respectively. The high seroincidences indicate that the underlying transmission dynamics of *S*. Typhimurium and *S*. Enteritidis infections in the Vietnamese infant population are far greater than estimated in prior studies [19, 21]. Therefore, public health control measures, potentially including vaccination and improved farm biosafety, are required to reduce transmission of *S*. Typhimurium and *S*. Enteritidis in this vulnerable age group [40, 41].

Our investigation also suggests that maternally acquired antibodies offer protection from NTS infections during the first months of infant life. Similar observations have been made in a Malawian pediatric cohort, which attributed limited invasive NTS disease during early infant life to transplacentally acquired antibodies [9, 18, 42]. Furthermore, our time-trend analysis showed that infants in urban Vietnam were most vulnerable to NTS infections (ie, the point of lowest immunity against *S*. Typhimurium and *S*. Enteritidis offered by maternally acquired antibodies) between 20 and 30 weeks of age. Estimation of the lowest passive immunity to *S*. Typhimurium and *S*. Enteritidis at the population level provides vital information for scheduling the delivery of future NTS vaccines, as has been the case with several successfully licensed infant vaccines [33, 43].

Although the majority of NTS disease cases occur during the first and second years of life [9], infants aged <1 year experience the greatest incidence of bacteremia-related deaths in Kenya and compose the age group that would benefit the most from NTS vaccination [44]. Therefore, current opinion is that vaccines against *S*. Typhimurium and *S*. Enteritidis should be given at 2–4 months of age [5]. However, polysaccharide vaccines in children aged <2 years elicit only weak antibody responses, and even conjugated polysaccharide vaccines induce poorer immune responses in neonates than in older children [45–47]. Maternal immunization during pregnancy is becoming an accepted strategy to protect infants from life-threatening infections such as tetanus, pertussis, and influenza [11, 48]. Taking into account observations from this study and existing evidence, we propose maternal immunization during pregnancy as a sensible strategy to prevent NTS disease during infancy and to delay infant immunization until 9 or 12 months of age, when it can be easily integrated with the existing Expanded Program on Immunization schedule.

Our study had several limitations. We were unable to estimate cumulative seroincidences for *S*. Typhimurium and *S*. Enteritidis infections, since some participating infants were lost to follow-up and therefore not followed until 1 year of age. Furthermore, only a maximum of 4 longitudinal plasma samples were collected from participating infants over the span of 1 year; this sampling frequency may have induced inaccuracy in the time-trend models. Additionally, although other group B and D *Salmonella* organisms are less common than *S*. Typhimurium and *S*. Enteritidis in Vietnam [49], the cross-reactivity from other *Salmonella* bacteria within the same group may introduce error.

NTS are associated with millions of infant cases of gastroenteritis and bacteremia globally, and mortality rates are likely to worsen as a consequence of antimicrobial resistance against key antimicrobials [50]. There are currently no human-approved NTS vaccines, although several are in the preclinical phase [5]. Our seroepidemiologic investigation defines the importance of maternally acquired antibodies for neonatal protection and suggests that the extent of NTS circulation and infant exposure in Vietnam is substantially larger than previously estimated [19]. Our study provides evidence of high NTS transmission in this setting and proposes maternal immunization as a potential control strategy against NTS disease in infants.

## Supplementary Data

Supplementary materials are available at *The Journal of Infectious Diseases* online. Consisting of data provided by the authors to benefit the reader, the posted materials are not copyedited and are the sole responsibility of the authors, so questions or comments should be addressed to the corresponding author.

## Supplementary Material

Supplementary Material

## Notes

***Acknowledgments.*** We thank the study participants and their families, without whom this study would not have been possible; the Dengue group at Oxford University Clinical Research Unit (OUCRU), for assistance in study design; all members of the enteric infections group at OUCRU; the study nurses and team at Hung Vuong Hospital; and the Clinical Trial Unit and Data Management Centre at OUCRU.

***Disclaimer.*** The funders had no role in study design, data collection and analysis, decision to publish, or preparation of the manuscript.

***Financial support.*** This work was supported by the Wellcome Trust and the Royal Society (Sir Henry Dale Fellowship 100087/Z/12/Z to S. B.).

***Potential conflicts of interests.*** All authors: No reported conflicts. All authors have submitted the ICMJE Form for Disclosure of Potential Conflicts of Interest. Conflicts that the editors consider relevant to the content of the manuscript have been disclosed.
