# Supplementary material for: The Role of Maternally Acquired Antibody in Providing Protective Immunity Against Nontyphoidal Salmonella in Urban Vietnamese Infants: A Birth Cohort Study
Source: J Infect Dis. 2018 Oct 12;219(2):295–304. doi: 10.1093/infdis/jiy501 (PMC6306017; doi:10.1093/infdis/jiy501)
Supplement: Supplementary Material [file jiy501_suppl_supplementary_material.docx]

**Supplementary Text 1: Samples and PCR for O-antigen ELISA validation**

Enzyme-linked immunosorbent assays (ELISA) for the identification of *S.* Typhimurium and *S.* Enteritidis exposure with the respective O-antigens (i.e. O4 and O9 antigens, respectively) were validated using acute-convalescent pairs of plasma samples from fecal PCR-positive *S.* Typhimurium and *S.* Enteritidis cases. These samples originated from a previously published study of pediatric patients presenting to hospital with severe diarrhea, where an acute blood sample and a fecal sample were collected on the day of hospital admission and a convalescent blood sample was taken at least 28 days later.[1]

Stool samples culture-positive for *Salmonella* groups B or D were taken from diarrheal patients described above and subjected to PCR for identification of *Salmonella* serovars. Several multiplex PCRs were established to screen for 1) *S.* Typhimurium, 2) *S.* Enteritidis, and several other common serotypes of 3) group B (i.e. *S.* Paratyphi B and *S.* Stanleyville) and 4) group D NTS (i.e. *S.* Dublin). DNA was extracted by suspending a single colony in 100μl of sterile water and subjecting it to 100^o^C for 10 min. Three μl of supernatant was PCR amplified for 15 min at 95°C, 35 cycles of 30s at 95°C, 30s at 63°C and 30s at 72°C, followed by 7 min at 72°C. The PCR primers used and their target regions are described in Table S1 in Supplement. Other NTS cases were defined as patients who were fecal culture-positive for *Salmonella* but PCR-negative by the specific *S.* Typhimurium and *S.* Enteritidis PCR. Fecal cultures positive for *Shigella* were used as acute *Salmonella* negative controls.

**Supplementary Text references**

1. Duong VT, Tuyen HT, Minh PV, Campbell J, Phuc HL, Nhu TDH, et al. No Clinical Benefit of Empirical Antimicrobial Therapy for Pediatric Diarrhea in a High-Usage, High-Resistance Setting. Clin Infect Dis. 2017; cix844.

**Supplementary Table 1**. Primers and target region for multiplex PCRs used to identify infecting Group D and B *Salmonella* serovars isolated from diarrheal patients

| **Serogroup** | **Serovar** | **Target** | **Primer name** | **Sequence (5’-3’)** | **Amplicon size (bp)** | **Ref** |
| --- | --- | --- | --- | --- | --- | --- |
| Group D | Enteritidis | Sdf I | sdfF | TGTGTTTTATCTGATGCAAGAGG | 293 | (1) |
|  |  |  | sdfR | CGTTCTTCTGGTACTTACGATGAC |  |  |
|  | Dublin | FliC-g,p | H-for | ACTCAGGCTTCCCGTAACGC | 779 | (2) |
|  |  |  | Hgp | ATTAACATCCGYCGCGCCAA |  |  |
| Group B | Typhimurium | FliB | Sense-59 | CAACAACAACCTGCAGCGTGTGCG | 1389 | (3) |
|  |  |  | Antisense-43 | GCCATATTTCAGCYTCTCGCCCG |  |  |
|  |  | FliA/B | FFLIB | CTGGCGACGATCTGTCGATG | 250/1000 | (4) |
|  |  |  | RFLIA | GCGGTATACAGTGAATTCAC |  |  |
|  | Paratyphi B | SPAB_01124 | pBP23-F | ACATAATGCTTTTCGTGCTCCTC | 388 | (5) |
|  |  |  | pBP23-R | GGCATAAATATCTTTCTCCCCTCC |  |  |
|  | Stanleyville | FliC-z24,z23 | Hz4,z23F | TTTGTCAAAGATGTTACTGCG | 428 | (2) |
|  |  |  | Hz4,z23R | AGGTTAGTGATGGCAGATTC |  |  |

(1) Agron P.G et al, Appl Environ Microbiol (2001) 67, 4984–4991

(2) Tennant S. at al, PLoS Neglected Tropical Diseases (2010), 4(3), e621

(3) Vanegas RA et al, J Bacteriol (1995) 177: 3863–3864

(4) Echeita MA et al, J Clin Microbiol (2001) 39: 2981–2983

(5) Zhai L et al, FEMS Microbiol Lett (2014), 355(1):83-9

**Supplementary Figure 1**. Anti-O4 and anti-O9 specific IgG antibody fold changes following natural infections by non-typhoidal *Salmonella* (NTS).

Fold change in IgM (A) and IgG (B) to O4 antigen was assessed following natural NTS infections. Fold change in IgM (C) and IgG (D) to O9 antigen was also assessed following natural NTS infections. Multiplex PCR was used to characterize the infecting *Salmonella* serovars and *Shigella*. Other group B infections were associated with the *Salmonella* serovars, Paratyphi B and Stanleyville, while other group D infections were associated with the *Salmonella* Dublin. Mann-Whitney *U* test was used to statistically compare groups, where * for 0·01<*p*≤0·05, ** for 0·001≤*p*≤0·01 and *** for *p*<0·001.


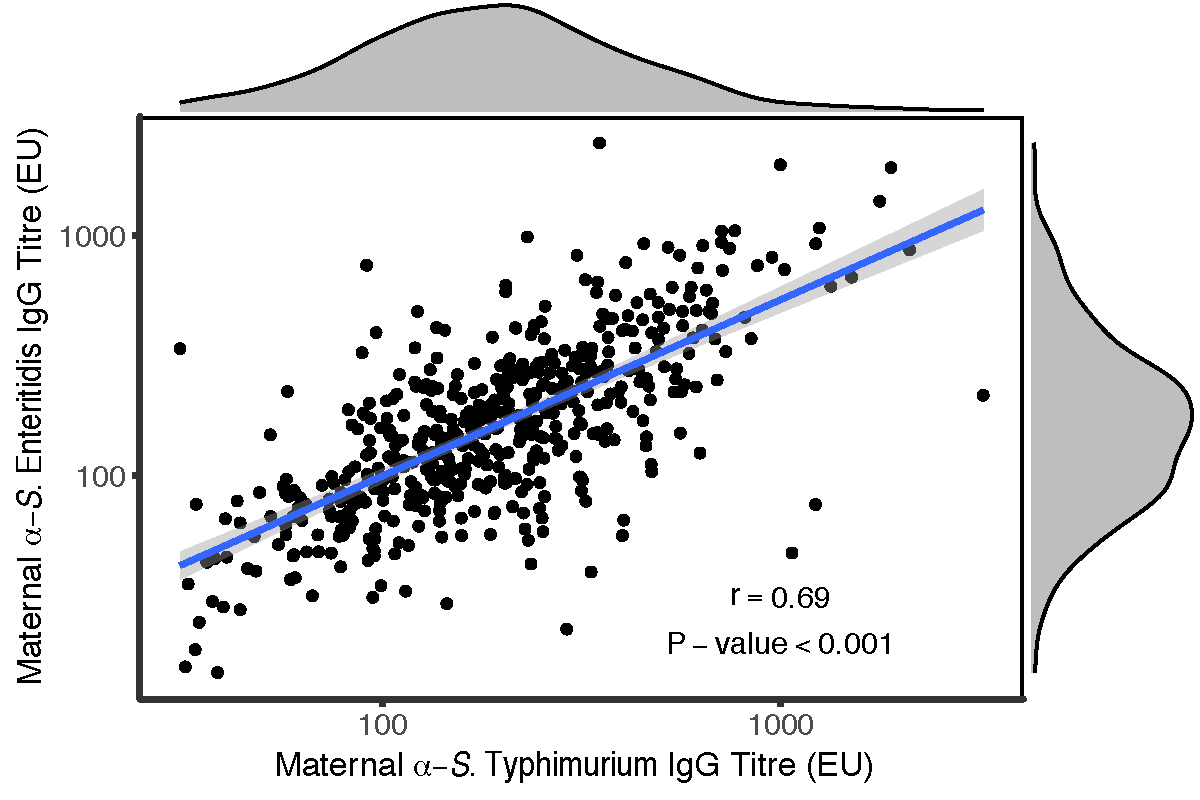


**Supplementary Figure 2**. Positive correlation observed between anti-*S*. Typhimurium antibodies (i.e. O4-specific IgG) and anti-*S*. Enteritidis antibodies (i.e. O9-specific IgG) in maternal plasma. Correlation between log10-transformed anti-O4 and anti-O9 IgG titer in the maternal samples was tested using the Pearson correlation test.


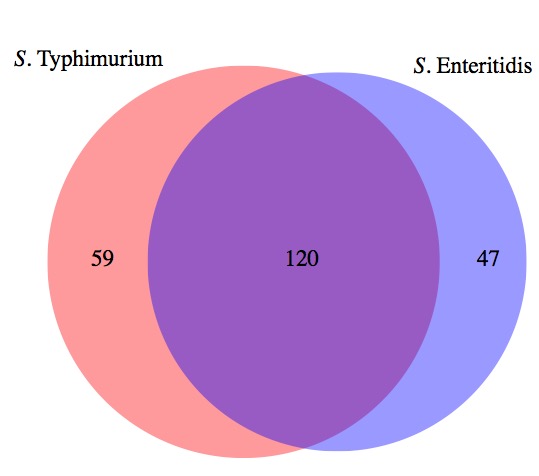


**Supplementary Figure 3**. Seroconversions to *S.* Typhimurium (i.e. O4 antigen) and *S.* Enteritidis (i.e. O9 antigen) antigen during the first year of life.

Venn diagram showing the number of seroconversions to *S.* Typhimurium (n=59), *S.* Enteritidis (n=47), and both antigens (n=120) during the first year of life.
